# Ultrasensitive Luteolin Electrochemical Sensor Based on Novel Lamellar CuZn@ Nitrogen-Containing Carbon Nanosheets

**DOI:** 10.3390/nano13010171

**Published:** 2022-12-30

**Authors:** Yuhong Li, Yaqi Yang, Jiejun Li, Li Zhang, Pengcheng Zhao, Junjie Fei, Yixi Xie

**Affiliations:** 1 Key Laboratory of Environmentally Friendly Chemistry and Applications of Ministry of Education, College of Chemistry, Xiangtan University, Xiangtan 411105, China; 2 Key Laboratory for Green Organic Synthesis and Application of Hunan Province, Xiangtan University, Xiangtan 411105, China; 3 Hunan Institute of Advanced Sensing and Information Technology, Xiangtan University, Xiangtan 411105, China

**Keywords:** lamellar structure, shape control, nitrogen-containing carbon, fast response

## Abstract

The Cu/Zn-zeolitic imidazolate framework (Cu/Zn-ZIF) was synthesized using the traditional hydrothermal method, and its surface morphology was controlled by adding polyvinylpyrrolidone (PVP) during its synthesis. It was then calcined at 800 °C to form the nitrogen-containing carbon material CuZn@NC, which improved the electron transfer rate. Scanning electron microscopy (SEM), X-ray crystal diffraction (XRD), and X-ray photoelectron spectroscopy (XPS) were used to investigate the surface morphology and structure. Finally, the electrochemical sensing platform for luteolin was effectively constructed by changing the metal–ion ratio during synthesis to achieve the most suitable electrode material. The sensor platform detects luteolin well, with an operating curve equation of Ip (A) = 0.0571C (nM) − 1.2913 and a minimum detection limit of 15 nM, and the platform has been successfully employed for luteolin detection in real samples.

## 1. Introduction

Luteolin is a naturally occurring flavonoid found in fruits and vegetables, natural herbs, olive oil, and other common foods, and it exhibits numerous excellent pharmacological activities in medicine, including anti-cancer, anti-bacterial, anti-inflammatory, expectorant, anti-spasmodic, anti-allergic, and immune-enhancing properties [1,2,3]. It is commonly utilized in clinical cough, expectorant, and anti-inflammatory medications [4,5]. As a result, it is critical to develop luteolin detection methods. At the present, luteolin detection techniques include high-performance liquid chromatography [6,7], gas chromatography [8], spectrophotometry [9], capillary electrophoresis [10], and electrochemical sensor methods [11,12], the latter of which is rapidly developing due to its fast response time, simple operation, and low cost. Furthermore, luteolin has four hydroxyl groups, which are easily oxidized, in its molecular structure, and the corresponding electrochemical response signal is strong, making it suitable for use in electrochemical sensors [13].

Luteolin electrochemical sensors have been studied in previous research; for example, Wang et al. [14] constructed an effective luteolin sensor by combining a Co-doped NC framework and multi-walled carbon nanotubes; Chen et al. [15] built a well-performing electrochemical sensor with a poly (3, 4-ethylene dioxythiophene) (PEDOT) and -cyclodextrin metal-organic framework (CD-MOF); and Zhang et al. [16] developed a luteolin detection platform based on hollow cobalt sulfide polyhedron–multi-walled carbon nanotube nanocomposites (CoSx–MWCNTs) and graphene quantum dots (GQDs). All of these previous study findings have been successful; however, their synthesized sensor materials are extremely restricted and unstable for practical applications [17]. As a result, there is an urgent need to create electrochemical sensor materials that are appropriate for mass manufacturing, with high performance and stability. Previous research has found that electrochemical sensor materials with good performance should have the following characteristics [18,19,20]: (1) good electrical conductivity and stability, (2) high specific surface area, (3) intrinsic active sites, and (4) well-defined structure and morphology.

In recent years, metal–organic frameworks (MOFs) with polymetallic nodes have been employed as excellent precursors for the fabrication of electrochemical sensor materials [21,22,23]. MOF can be turned into N-doped carbon materials (NC) with strong electrical conductivity and high specific surface area through easy pyrolysis, while their morphological structure remains orderly, and in which the metal nodes can be reduced to metal nanoparticles, which can then be inserted into the N-doped carbon materials, revealing their intrinsic active sites [24,25]. However, metal@NC (M@NC) materials produced from monometallic MOFs are still insufficient for electrochemical sensors. Preparing bimetallic MOFs by inserting new metal centers is a potentially viable option for increasing the material’s electrochemical activity by leveraging the synergistic effect of the bimetals and better controlling the morphological structure of the MOF [18,26]. For example, Wang et al. [27] explored the application of bimetallic zeolitic imidazolate framework (BM-ZIF) derivatives of 2-Methylimidazole zinc salt (ZIF-8) and 2-Methylimidazole cobalt salt (ZIF-67) for circulating deionization capacitors, and Liu et al. [28] constructed a novel uric acid electrochemical sensor based on ZIF-8 and ZIF-67 bimetallic ZIF, with a linear range of 2.0 μM~110 μM and a low detection limit of 0.83 μM at S/N = 3. Guan et al. [29] applied Cu/Zn-ZIF-derived carbon materials as cell materials for zinc-air batteries. These experiments fully demonstrated the material’s application potential.

Based on the previous findings, Cu/Zn bimetallic MOF-derived nitrogen-doped carbon materials (CuZn@NC) were synthesized in this research, and their surface shape was regulated to exhibit a lamellar morphology, thereby increasing the specific surface area and exposing more active sites. The sensor demonstrated strong luteolin detection and selectivity, owing to the material’s lamellar structure and bimetallic catalytic site; in addition, the minimum detection limit of the sensor was calculated to be 15 nM for the detector using the DPV method, and the sensor was successfully used to detect luteolin in real samples. This method could lead to new applications for luteolin in the field of food diagnostics.

## 2. Materials and Methods

### 2.1. Reagents and Equipment

C_15_H_10_O_6_ (luteolin) was obtained from Shanghai Yuanye Bio-Technology Co., Ltd., (Shanghai, China). Cu(NO_3_)_2_, Zn(Ac)_2_, PVP, 2-methylimidazole (2-MI), and NaCl were provided by Aladdin Reagent Co., Ltd. (Shanghai, China). C_2_H_5_OH, Na_2_HPO4, and NaH_2_PO_4_ were purchased from Hunan Huihong Reagent Co., Ltd., (Chengdu, China). Standard phosphate buffer solutions (PBS), with pH values ranging from 5.0 to 8.5, were produced by mixing sodium dihydrogen phosphate (0.1 M NaH_2_PO_4_) with sodium monohydrate phosphate (0.1 M Na_2_HPO_4_). All of the compounds used were of analytical quality and were not purified further.

During material synthesis, CuZn@NC was annealed in a muffle furnace (MFLGKDF 206-12, China). The morphology and structure of CuZn@NC were characterized by scanning electron microscopy (SEM) (JSM-6610LV, Japan), X-ray diffraction (XRD) (Bruker D8 Discover, Germany) and X-ray photoelectron spectrometer (XPS) (Thermo Fisher ESCALAB 250Xi, Waltham, MA, USA). The electrochemical characterization equipment consisted of a CHI-660E electrochemical workstation (Shanghai Chenhua Instruments Co., Ltd., Shanghai, China) with a three-electrode system that included GCE (bare or modified) as the working electrode, Pt wire as the counter electrode, and Ag/AgCl as the reference electrode.

### 2.2. Synthesis of Cu/Zn-ZIF and CuZn@NC

According to previous research [29], 482 mg of Cu(NO_3_)_2_ (2 mM) and 876 mg of Zn(Ac)_2_ (4 mM) were dissolved in 40 mL of deionized water (A solution), and then 1312 mg of 2-MI and 500 mg of PVP were dissolved in 40 mL of water (B solution), and the AB solution was thoroughly mixed and then heated in oil. After the reaction, the solution was centrifuged at 8000 rpm for 15 min, washed 4 times with deionized water, and lastly dried under vacuum at 60 °C overnight. Zn ratios of 1:2, 1:1, and 2:1 were named Cu/Zn-ZIF-1, Cu/Zn-ZIF-2, and Cu/Zn-ZIF-3, respectively.

The resulting Cu/Zn-ZIF was thoroughly crushed before being placed in a muffle furnace and heated to 800 °C at a rate of 3 °C·min^−1^ for 2 h. The product, CuZn@NC, was obtained after natural cooling. CuZn@NC was disseminated in DMF to create a suspension of 0.5 mg·mL^−1^.

### 2.3. Working Electrode Preparation of CuZn@NC/GCE

**Scheme 1:**
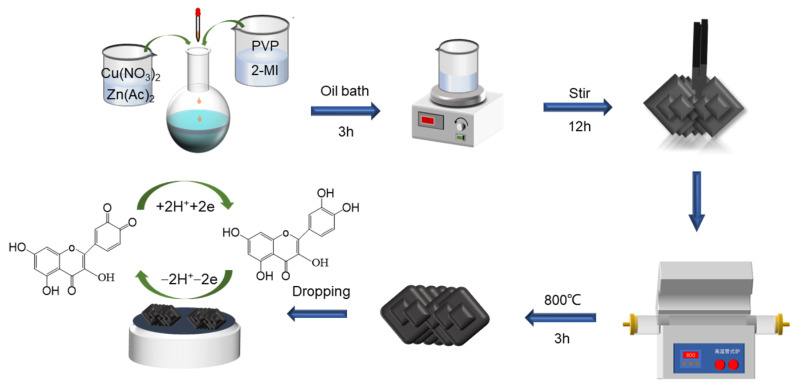
Schematic illustration of the fabrication of CuZn@NC/GCE.

The surface of GCE was first polished with alumina powder, then ultrasonically washed with ethanol and water, and the cleaned electrode was put under an infrared light for baking; lastly, 6 μL of CuZn@NC suspension was pipetted on the surface of GCE with a pipette gun, and the liquid beads on the surface of the electrode were baked dry using an infrared warming lamp to obtain a functioning electrode CuZn@NC/GCE that could be utilized for testing. Scheme 1 depicts the preparatory procedure.

### 2.4. Preparation of Actual Samples

Luteolin can be found in a variety of plants and foods in nature. To obtain the actual luteolin sample solution, we purchased a batch of freeze-dried chrysanthemum tea online and soaked one piece of chrysanthemum (0.352 g) in 60 mL of anhydrous ethanol for 3 h. To measure the amount of luteolin in the actual sample test, 100 μL of chrysanthemum solution was added to 0.1 M PBS.

### 2.5. Electrochemical Test Parameters

The cyclic voltammetry (CV) potential range was set between −0.2 V and 0.8 V, with a sweep rate of 50 mV·s^−1^, and the differential pulse voltammetry (DPV) potential range was set between 0.2 V and 0.5 V. In a three-electrode setup, clean platinum wire electrodes were utilized as counter electrodes, while electrodes filled with saturated silver chloride solution were employed as reference electrodes. Test investigations were carried out in 0.1 M PBS solution containing sodium chloride at a concentration of 0.05 M. Electrochemical impedance measurements were carried out in a solution of 1.0 mM [Fe(CN)_6_]^4−^/^3−^ containing 0.1 M KCl, with the amplitude set at 50 mV, the pulse width set at 5 mV, and the frequency range set between 100 kHz and 0.01 Hz.

## 3. Results and Discussion

### 3.1. Physical and Electrochemical Characterization

**Figure 1:**
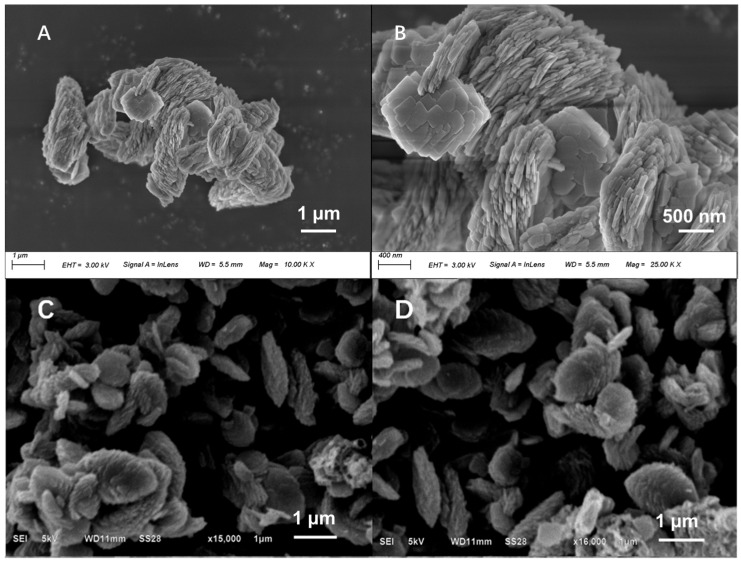
SEM images of CuZn-ZIF (**A**,**B**); CuZn@NC (**C**,**D**).

Figure 1 depicts the surface morphology of several materials at different resolutions; Figure 1A,B show the morphology of Cu/Zn-ZIF, which demonstrates a multilayered and loose lamellar structure; the calcined material CuZn@NC still exhibits a lamellar scale-like structure (Figure 1C,D), but it has a more constant shape and a homogeneous texture, and the structure is not substantially collapsed, as it was before calcination. The surfactant PVP introduced during synthesis regulated and reduced the surface morphology of the MOF well, allowing it to eventually display this structure and expand the electrode material’s surface area.

**Figure 2:**
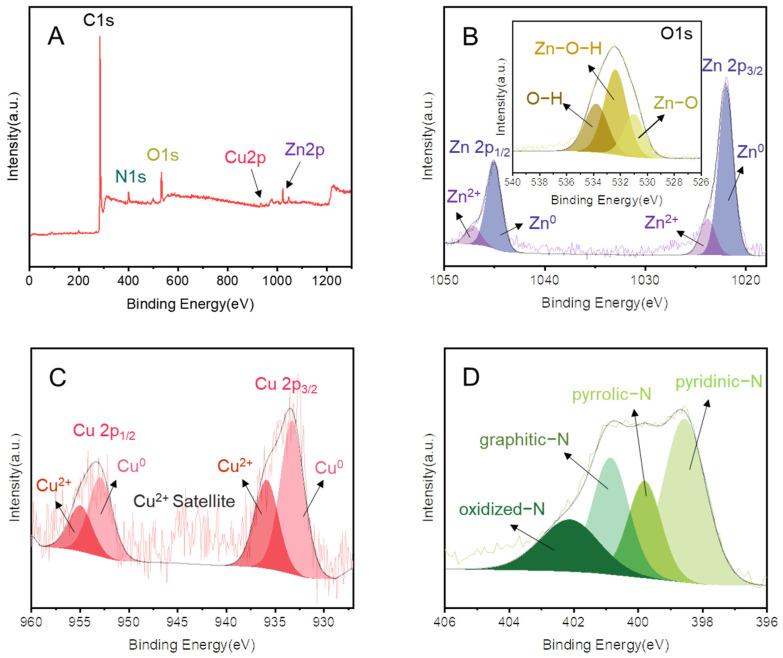
XPS survey (**A**), Zn 2p (inset: O 1s) (**B**), Cu 2p (**C**), and N 1s (**D**) spectrum of CuZn@NC.

XPS was used to analyze the surface composition and chemical state of CuZn@NC, and the spectral peaks were calibrated with C 1s (284.80 eV) [30]. The spectral peaks of C 1s, Cu 2p, Zn 2p, O 1s, and N 1s are shown in Figure 2A. The Zn 2p peaks are divided into Zn 2p3/2 (1021.98 eV and 1023.78 eV), and Zn 2p1/2 (1045.08 eV and 1047.08 eV), where the binding energies of 1021.98 eV and 1045.08 eV correspond to Zn^0^, and 1023.78 eV and 1047.08 eV correspond to Zn^2+^ [31,32,33]. In addition, the built-in diagram shows three splitting peaks of O1 at 530.98 eV, 532.38 eV, and 533.78 eV, corresponding to Zn-O, Zn-O-H, and O-H, respectively [34,35]. Figure 2C shows two distinct splitting peaks at Cu 2p_3/2_ (933.23 eV, 952.9 eV) assigned to the metal Cu^0^, demonstrating that the metal Cu is effectively reduced, while the splitting peaks at Cu 2p_1/2_ (935.86 eV, 954.95 eV) belong to Cu^2+^ (CuO), and the heterogeneous peaks between Cu 2p_3/2_ and Cu 2p_1/2_ are Cu^2+^ Satellite [36,37,38]. Figure 2D depicts N 1s splitting at 398.58 eV (pyridinic-N), 399.78 eV (pyrrolic-N), 400.88 eV (graphitic-N), and 402.18 eV (oxidized-N), indicating that the N elements were evenly doped with C elements during high-temperature calcination. These findings imply that the CuZn@NC synthesis was effective. The parameters used for the XPS peak fit are shown in Table 1.

**Figure 3:**
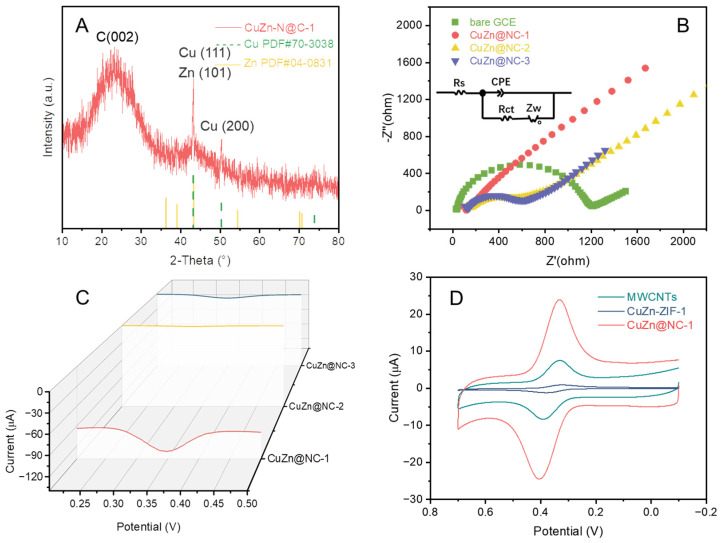
(**A**) XRD patterns of CuZn@NC, (**B**) EIS spectra of different electrode materials, supporting electrolyte solution: 1 mM Fe (CN)_6_^3-/4-^ (containing 0.1 M KCl), frequency range: 0.01 Hz to 100 kHz, (**C**) DPV, and (**D**) CV electrochemical responses of different electrode materials.

XRD was used to analyze the crystal structure of CuZn@NC. Figure 3A displays the polycrystalline structure of CuZn@NC, which reveals a distinctive peak at 2θ = 25.73°, which is compatible with the characteristic peak of graphene at 2θ = 25.73°(002). The characteristic peak at 2θ = 43.23°, which corresponds to the diffraction planes of Zn (101) (PDF#04-0831), 2θ = 43.19° and 2θ = 50.3°, which corresponds to the diffraction plane of Cu (111) and Cu (200) (Cu PDF#70-3038); the structures are in general agreement with those described in the literature; thus, it can be proved that CuZn@NC has been successfully prepared.

CuZn@NC-1, CuZn@NC-2, and CuZn@NC-3, with different metal ratios, were modified on the electrode surface and tested in 1 mM Fe(CN)_6_^3−^/^4−^ (containing 0.1 M KCl) solution, and the electrochemical impedance spectroscopy (EIS) was used to characterize the charge transfer resistance (Rct) on the surface of several modified electrodes, and the electrochemical model (Rs((Rct-Zw)-CPE)) was fitted to obtain the Rct values. In general, the lower the Rct and the higher the conductivity, the smaller the radius of the curve. As can be seen from Figure 3B, the impedance of bare glass is the largest (Rct = 1132 Ω), and CuZn@NC-2 (Rct = 581 Ω) and CuZn@NC-3 (Rct = 500.5 Ω) are slightly smaller, but the radius of the curvature of the curve is still relatively large, while the radius of the curvature of the curve of CuZn@NC-1 is the smallest, nearly becoming a straight line. This might be because the Cu/Zn ratio of CuZn@NC-1 is better suited to exploiting the bimetal’s synergistic effect, resulting in excessively low electron transfer impedance at the electrode surface. This also implies that the CuZn@NC-1 electrode surface has the fastest electron transfer rate [39,40].

The electrode mentioned above was placed in a PBS buffer solution at pH = 5.5 and punched with 500 nM luteolin for the DPV test; the test results are shown in Figure 3C, and it can be noted that CuZn@NC-1 has the best electrochemical response to luteolin, CuZn@NC-3 ranks second, and CuZn@NC-2 is the worst. Combined with the literature analysis, it is thought that this is because CuZn@NC-2 and CuZn@NC-3 formed small metal beads and detached from the material during calcination, causing the number of metal active sites in the electrode material to decrease and triggering the decrease in the electron transfer rate; therefore, CuZn@NC-1 was chosen as the main electrode modification material for testing in this paper. Finally, MWCNTs were modified on the glassy carbon electrode, and 3 μM luteolin was punched into the PBS buffer at pH = 5.5 for CV testing (Figure 3D) Compared with CuZn@NC-1 and bare GCE, it can be found that the electrochemical response of CuZn@NC-1 to luteolin is twice as high as that of MWCNTs, and significantly larger than that of CuZn-ZIF-1 before calcination. This proves that the metal active site in CuZn@NC-1 plays a catalytic role and amplifies the electrical signal.

**Figure 4:**
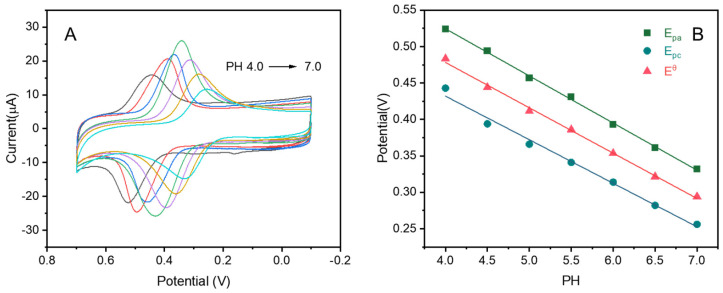
(**A**) CV curves of 3 μM luteolin on CuZn@NC−1/GCE with different pHs; (**B**) linear relationship between oxidation potential (E_pa_), standard electrode potential (E^θ^), reduction potential, and pH (E_pc_).

CuZn@NC-1/GCE was placed into PBS buffer solutions containing 3 μM luteolin at different pHs (4.0, 4.5, 5.0, 5.5, 6.0, 6.5, 7.0) to test the electrochemical signals at different pH conditions, as can be seen from the CV superimposed plots in Figure 4A; in the interval from pH = 4.0 to 5.5, the redox peak current of luteolin increased with the increase in pH and reached the maximum value at a pH equal to 5.5, while the redox peak current of luteolin began to gradually decrease when the pH continued to change from 5.5 to 7.0. It can be seen that the optimal pH value of CuZn@NC-1/GCE for luteolin detection is 5.5, and we will choose pH = 5.5 for subsequent experiments.

We also studied the influence of pH on the redox peak potential value. In Figure 4A, the redox peak potential value is shifted to the right as the solution pH gradually approaches neutrality. Graphing the relationship between pH and peak potential value, it is also found that there is a linear relationship between pH and peak potential value in the pH range of 4.0–7.0 (Figure 4B), with the linear equation:

E_pa_ = −0.0647pH + 0.7834 (R² = 0.9988)

E_pc_ = −0.0598pH + 0.6711 (R² = 0.9905);

E^θ^ = −0.0622pH + 0.7272 (R² = 0.9976)

The coefficients of the above three equations are very close to the theoretical value of 59 mV/pH given by the Nernst equation, and this result indicates that the number of electrons transferred during the reaction of luteolin at the surface of the CuZn@NC-1/GCE electrode is equal to the number of protons involved in the reaction, which is an “equal-electron–equal-proton” process [41,42].

**Figure 5:**
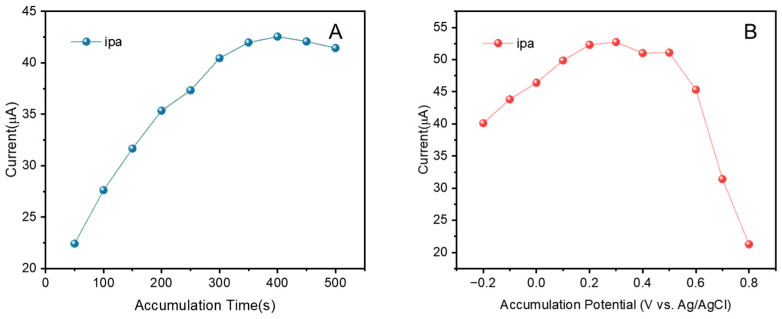
CuZn@NC-1/GCE oxidation current values vs enrichment time (**A**) and enrichment potential (**B**) in PBS buffered solution with PH = 5.5 and 1 μM luteolin.

In light of the previous results, we decided to use the DPV approach to evaluate the optimal enrichment parameters for the electrochemical response to luteolin with 1 μM luteolin at pH = 5.5. Figure 5A shows the optimum peak current findings after optimizing the enrichment time. When the enrichment time was increased from 50 s to 400 s, the electrochemical response signal of luteolin increased with it, and after 400 s, the electrochemical response signal of luteolin decreased slightly, indicating that luteolin adsorption on the electrode surface had reached its maximum value, achieving a saturation state. As a result, we determined that 400 s was the best time for luteolin enrichment. As shown in Figure 5B, the electrochemical response signal of luteolin steadily rose as the enrichment potential was increased from −0.2 V to 0.3 V, using the same approach. The peak current began to diminish as the migration from 0.3 V to 0.8 V continued, and although it marginally returned at 0.5 V, it was still not as robust as the electrochemical response signal when the enrichment potential was 0.3 V. From 0.5 V to 0.8 V, the electrochemical response signal rapidly decreases from 0.5 V to 0.8 V. Thus, the optimal enrichment potential for measuring luteolin was chosen as 0.3 V.

**Figure 6:**
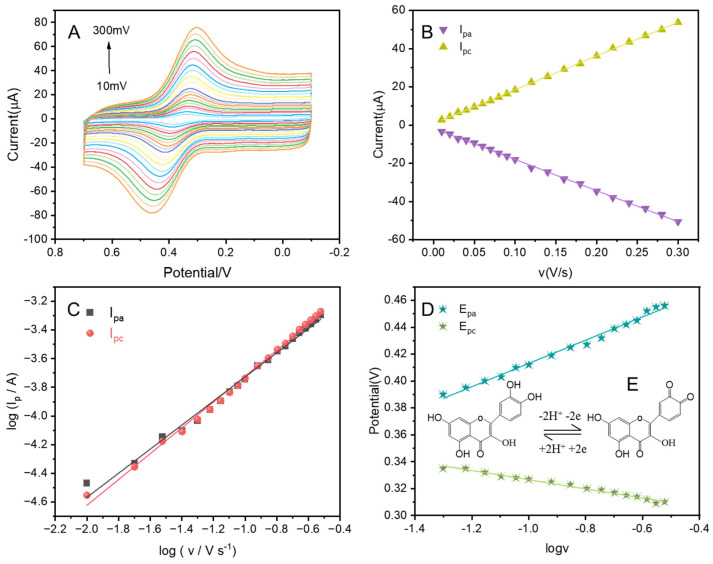
(**A**) CuZn@NC−1/GCE CV superposition at different sweep speeds in a PBS solution containing 3 μM luteolin; (**B**) graph depicting the link between the sweep rate and the oxidation and reduction peak currents; (**C**) logarithmic relationship between peak current and sweep speed; (**D**) plot of the relationship between the logarithm of the peak current and the potential; (inset: (**E**)) possible electrochemical oxidation reaction of luteolin.

Based on the optimization described above, the electrochemical behavior of luteolin on CuZn@NC-1/GCE electrodes was investigated at various scan speeds. Figure 6A shows that when the scan rate increases, the oxidation potential (E_pa_) swings to a positive potential, and the reduction potential (E_pc_) shifts to a more negative potential, showing that this is a reversible redox process. Figure 6B clearly shows that the oxidation peak current (I_pa_) and reduction peak current (I_pc_) have a highly correlated linear relationship with the scan rate, implying that the reversible redox reaction of luteolin on the modified electrode surface is an adsorption-controlled process, with the following linear equation: I_pa_ (μA) = 0.1768v (mV·s^−1^) + 0.7675 (R² = 0.9996); I_pc_(μA) = −0.1631v (mV·s^−1^) − 1.6811 (R² = 0.9992)

Previous research has shown that a slope value can be calculated from the logarithmic relationship between peak current and sweep speed [43,44,45]; if the slope is close to 0.5, the reaction is diffusion-controlled; if the slope is close to 1, the reaction is adsorption-controlled, and the slope values calculated from the relationship diagram in Figure 6C are all 0.84, which is close to 1. y = 0.8408x − 2.886 (R^2^ = 0.9902) is the linear connection. This finding confirms that the luteolin redox reaction on the modified electrode surface is an adsorption-controlled mechanism.

To obtain the charge transfer coefficient (α) and the number of electron transfers (n) for this reaction, consider the relationship between log v and E (V) [46], as shown in Figure 6D, and the equation is E_pa_(V) = 0.0865 log ν(V·s^−1^) + 0.4996 (R^2^ = 0.9911); E_pc_ (V) = 0.0341 log ν (V·s^−1^) + 0.2925 (R^2^ = 0.9857). When combined with the Laviron equation, we obtain α = 0.717, n = 2.39, and the charge transfer number of the reaction is 2. Figure 6E (inset) depicts one probable process.

### 3.2. Quantitative Analysis of Luteolin on CuZn@NC-1/GCE

**Figure 7:**
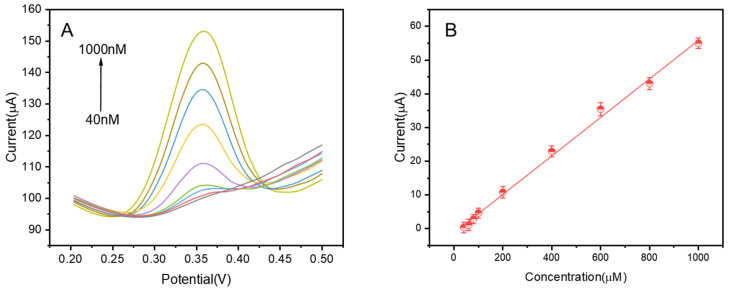
(**A**) A DPV overlaid map of luteolin on the CuZn@NC-1/GCE; (**B**) a linear relationship between DPV oxidation current and luteolin concentration.

After all of the above optimizations were accomplished, the DPV approach was used to estimate the working curve, linear range, and detection limit of this electrode. Figure 7A depicts the DPV stacked plots of the luteolin electrochemical response signal (40 nM, 60 nM, 80 nM, 100 nM, 200 nM, 400 nM, 600 nM, 800 nM, 1000 nM). The DPV response signal increases with increasing luteolin concentration, and we fit the electrochemical response signal, as well as the concentration, as shown in Figure 7B, and the equation for this linear relationship is presented below: Ip (μA) = 0.0571C (nM)-1.2913 (R² = 0.9963). The minimum detection limit was calculated to be 15 nM (S/N = 3), so we believe that the CuZn@NC-1/GCE electrochemical sensor for luteolin has been successfully constructed, the electrode material is relatively simple and easy to prepare, and most importantly, this provides a scheme for future research in the same direction.

Some reported sensors for luteolin are provided in Table 2 with data to help evaluate the performance of the current sensor. Among them, our study shows stronger performance and a lower detection limit, implying that CuZn@NC-1/GCE has good luteolin detection capability.

### 3.3. Interference Resistance, Reproducibility, and Stability Studies

**Figure 8:**
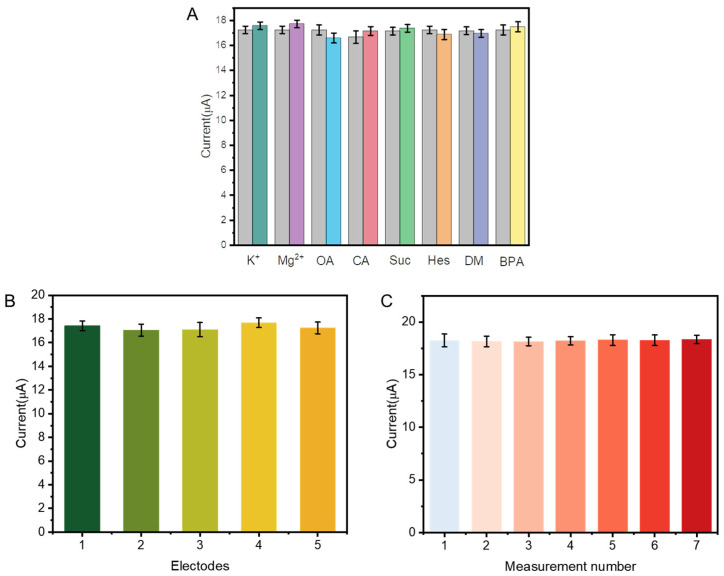
(**A**) DPV oxidation current in CuZn@NC-1/GCE in PBS containing various compounds, (**B**) DPV current obtained by 5 different electrodes in 300 nM luteolin, (**C**) DPV current obtained by 7 repeated tests in 300 nM luteolin.

This experiment was carried out to test the effect of interfering substances on luteolin detection by DPV. A total of 300 nM of luteolin was added in 0.1 M pH = 5.5 PBS buffer solution, and the electrochemical response signal of blank interference was detected first, followed by 50-fold concentrations of K^+^, Mg^2+^, oxalic acid (OA), citric acid (CA), sucrose (Suc), and 10-fold concentrations of hesperidin (Hes), diosmetin (DM), and bisphenol A (BPA). Figure 8A shows the histogram of the blank signal compared with the signal after the addition of the interferents. The experiments showed that when the interferents at the above concentrations were present, the floating range of the electrical signal of luteolin was within 5% of detection. This finding revealed that the electrochemical sensor platform developed in this experiment exhibits good luteolin selectivity and anti-interference properties.

The same approach was used to investigate 5 independent electrodes, and the peak currents of these 5 electrodes were compared to ensure consistency. Figure 8B depicts the experimental outcomes. The peak currents of luteolin on these 7 electrodes were essentially the same after calculation, and the RSD of the 5 sets of data was 2.37%, demonstrating that the electrode material exhibited essentially the same detection ability on any one glassy carbon electrode, with good reproducibility. After scanning 300 nM of luteolin with another electrode and waiting for the data to stabilize, the peak current data of 7 scans were acquired, and the experimental results are displayed in Figure 8C. The investigations revealed that the peak current of luteolin scarcely change in these 7 experiments, and the RSD of the 7 datasets was 0.55%, demonstrating that the CuZn@NC-1/GCE produced in this experiment shows good stability.

### 3.4. Testing of Actual Samples

The previously produced chrysanthemum solution was assayed, and the luteolin concentration in it was evaluated using the standard addition method under the optimal circumstances of the previous assay by first adding 100 μL of the chrysanthemum solution. The luteolin standard solution was then added at 50%, 100%, and 150% of the detected luteolin. The results are reported in Table 3, with recoveries in the real sample assay ranging from 94.7% to 95.9% and RSD between 0.28% and 0.32%, proving that the sensor platform may be employed for luteolin detection in real samples.

## 4. Conclusions

A luteolin electrochemical sensor platform CuZn@NC/GCE was built via a drop-coating approach using bimetallic MOF derivatives: Cu/Zn-ZIF. The resulting material possesses a lamellar squamous structure, which exposes more active sites and exhibits a greater specific surface area than other MOFs. The resulting carbon material has better electron transport capabilities than the initial metal-organic framework material. Before employing it as an electrode material, the best electrode material CuZn@NC-1/GCE was obtained by altering the ratio of different metals within the material. The electrode was very effective for detecting luteolin in 0.1 M pH = 5.5 PBS solution using DPV, and the electrical signal showed a linear relationship with the concentration at luteolin concentrations ranging from 40 nM to 1000 nM, with the linear equation Ip (A) = 0.0571C (nM) − 1.2913, and the resulting sensor shows good resistance to interferents in the solution to be measured. Furthermore, the sensor offers outstanding reproducibility and stability and has been successfully employed for luteolin detection in real samples.

## Figures and Tables

**Table 1:** All XPS signals information.

Name	Start BE	Peak BE	End BE	FWHM eV	Atomic %
C1s	297.98	284.68	279.18	1.64	86.28
Cu2p	964.98	932.98	925.18	2.57	0.36
N1s	409.98	399.14	392.18	4.02	5.01
O1s	544.98	532.33	525.18	3.57	7.51
Zn2p	1051.98	1021.91	1015.18	1.82	0.83

**Table 2:** Comparison of electrochemical properties of luteolin detected by different modified electrodes.

Luteolin Sensors	Detection Measures	Linear Range (μM)	LOD (nM)	Reference
Au/Pd/rGO/GCE	DPV	0.01–80	0.98	[47]
AuNCs/CILE	DPV	0.001–1	0.4	[48]
AR/f-MWCNT/GCE	i-T	0.5–45	170	[49]
UiO-66/ErGO/GCE	DPV	0.001–20	0.75	[50]
MWCNTs/PEDT-Au/GCE	SWV	0.1–15; 0.001–0.1 μM dm^−3^	0.22 μM dm^−3^	[51]
ZrO_2_/CS/rGOA/GCE	DPV	0.005–1	1	[5]
Co_9_S_8_/GCE	DPV	0.01–20	0.8	[52]
Nbim/CNT/GCE	DPV	0.005–0.32	0.6	[53]
Bare GCE	DPV	0.01–1	5	[54]
CuZn@NC/GCE	DPV	0.04–1	15	This work

**Table 3:** Detection of luteolin in chrysanthemum tea (n = 3).

Add (nM)	Detected (nM)	Recovery (%)	RSD (%)
0	60.0	---	---
30	88.9	96.3	0.32
60	116.8	94.7	0.46
90	146.3	95.9	0.28

## Data Availability

Data are contained within the article.
